# 

**Published:** 1964-07

**Authors:** 


					July 1904
\ NATIONAL LIBRARY OF MEDICINE
71, uf
RECC- SEP 2-3 1935
~r77-3y>3> VOL 'SSUE j
/<? IMDIiXER I
incorporating the medical journal of the south west
Vol 79 (iii) NO 293

				

